# The Wrong Spirit

**Published:** 1919-02-08

**Authors:** 


### THE WRONG SPIRIT.

A general atmosphere of restless indiscipline, an absence of fairness and sound judgment, and a loss of realisation of what is due to one's self-respect through self-discipline, threaten to impede the working and administration of some hospitals. This is much to be regretted. When taken with the exhibition of some of these undesirable qualities by those who attended the meeting at Wigmore Hall on Sunday last, it must cause all interested in the highest efficiency and sound welfare of the medical profession and the hospitals to feel regrettable surprise. Is there not at least one, or shall we say at least three, able administrators, who are members of the medical profession, practised in public affairs, whom the members can rally round, and under whose leadership they will combine? The hour glass is emptying rapidly, and it may soon be too late to save the profession and give it back the authority and the power to manage its affairs with ability and success. There is a sad lack of unity in the profession at the present time. Evidence of contention and disagreement has tended of late to increase to a lamentable and indeed a dangerous extent. The proceedings at Sunday's meeting cannot add to the dignity of the medical profession or its members. Unless practitioners as a body pull themselves together and once more rule and control in their own house, serious disaster must follow the patent evils of the moment.

It is further greatly to be regretted that there are signs of the absence of unselfish loyalty to colleagues and authorities. We have not space this week to treat in detail the extraordinary and regrettable proceedings which have arisen in connection with the Westminster Hospital. At Cardiff Colonel Bruce Vaughan, whose devotion and able development of King Edward VII. Hospital in that city have been the admiration not only of his neighbours, but of hospital administrators throughout the country, and his friends have been assailed in the Cardiff papers without just cause. This is to put in plain English the underlying meaning of the arguments used. Such unwarrantable action is reprehensible, and the charges lack justification or reasonable cause. Their ineptitude has, however, been raised to a high point by introducing as a hospital expert, of the highest repute, a most estimable man, who was an advocate, we believe, of the plan of the First Eastern General Hospital, Cambridge, of which, so long ago as our issue of December 16, 1916, we were impelled in justice to state: "We have never regarded this as one of the most successful attempts in temporary hospital construction which the war has produced. There were palpable errors in the planning of the original pavilions." Colonel Bruce Vaughan, good man as he is, has made a crushing reply to his detractors, who ought to apologise for the course they have taken. We are confident that the people of Cardiff will not be moved by attacks without real substance. They may be due, we understand, to a restless disloyalty on the part of some who should long ago have ceased to be connected with King Edward VII. Hospital, Cardiff.

